# Deep Learning-Based Object Detection Strategies for Disease Detection and Localization in Chest X-Ray Images

**DOI:** 10.3390/diagnostics14232636

**Published:** 2024-11-22

**Authors:** Yi-Ching Cheng, Yi-Chieh Hung, Guan-Hua Huang, Tai-Been Chen, Nan-Han Lu, Kuo-Ying Liu, Kuo-Hsuan Lin

**Affiliations:** 1 Institute of Statistics, National Yang Ming Chiao Tung University, Hsinchu 300093, Taiwan; 2 Department of Radiological Technology, Faculty of Medical Technology, Teikyo University, Tokyo 173-8605, Japan; 3 Infinity Co., Ltd., Taoyuan 320021, Taiwan; 4 Der Lih Fuh Co., Ltd., Taoyuan 320021, Taiwan; 5 Department of Radiology, E-Da Cancer Hospital, I-Shou University, Kaohsiung 824005, Taiwan; 6 Department of Emergency Medicine, E-Da Hospital, I-Shou University, Kaohsiung 824005, Taiwan

**Keywords:** chest X-rays, deep learning, few-shot object detection, object detection

## Abstract

Background and Objectives: Chest X-ray (CXR) images are commonly used to diagnose respiratory and cardiovascular diseases. However, traditional manual interpretation is often subjective, time-consuming, and prone to errors, leading to inconsistent detection accuracy and poor generalization. In this paper, we present deep learning-based object detection methods for automatically identifying and annotating abnormal regions in CXR images. Methods: We developed and tested our models using disease-labeled CXR images and location-bounding boxes from E-Da Hospital. Given the prevalence of normal images over diseased ones in clinical settings, we created various training datasets and approaches to assess how different proportions of background images impact model performance. To address the issue of limited examples for certain diseases, we also investigated few-shot object detection techniques. We compared convolutional neural networks (CNNs) and Transformer-based models to determine the most effective architecture for medical image analysis. Results: The findings show that background image proportions greatly influenced model inference. Moreover, schemes incorporating binary classification consistently improved performance, and CNN-based models outperformed Transformer-based models across all scenarios. Conclusions: We have developed a more efficient and reliable system for the automated detection of disease labels and location bounding boxes in CXR images.

## 1. Introduction

Medical images play a crucial role in disease prevention, detection, and diagnosis, providing essential support for clinicians. Of the various types, chest X-ray (CXR) images are particularly valuable for detecting abnormalities in the lungs, heart, and bones, which aids in making appropriate treatment decisions. The accurate analysis of these images is highly beneficial for improving patient care. In this study, we aim to enhance diagnostic accuracy by analyzing CXR images for 12 common chest conditions, including aortic sclerosis (calcification), arterial curvature, small pulmonary nodules, pulmonary nodule shadows, tuberculosis, pulmonary fibrosis, increased lung markings, prominent hilar regions, spinal lesions, intercostal pleural thickening, cardiac hypertrophy, and the presence of heart pacemakers. Twelve conditions were selected for study due to their reliable high-quality annotations, which are crucial for developing accurate models. Additionally, they have clear visual manifestations, making them more detectable via automated analysis.

Traditionally, doctors manually detect abnormalities in chest images through visual examination, which can be influenced by personal biases and external factors, leading to inconsistent results. During initial CXR screenings, physicians must also manually label lesion areas, a process that is time-consuming and labor-intensive. With the rapid growth in the volume of clinical image data, the workload for doctors has increased significantly. In recent years, artificial intelligence, particularly machine learning (ML), has emerged as a powerful tool for addressing such challenges. Deep learning, a subset of ML, has shown great success in computer vision tasks like image classification, segmentation, and object detection. Consequently, researchers have begun applying deep learning to medical image analysis to automate tasks such as disease diagnosis, detection, and lesion localization. These automated methods allow time savings, improving diagnostic efficiency and reducing the impact of external factors.

Several studies have applied deep learning to classify CXR images, aiming to aid in diagnosing a wide range of diseases. In particular, deep learning has been widely used to support automated diagnosis of COVID-19 from CXR images. For example, Ali et al. [1] developed a densely connected squeeze convolutional neural network (CNN) for classifying cases of COVID-19 and pneumonia with high accuracy, showcasing the potential of deep learning to enhance diagnostic reliability in the context of a pandemic. Singh et al. [2] also proposed a CNN architecture where segmentation and classification were combined to boost the classification accuracy for COVID-19-affected CXR images. Other studies have focused on identifying various types of pneumonia. Garstka and Strzelecki [3] developed a custom CNN, trained on a small dataset, to classify pneumonia types from CXR images. Additionally, recent studies have explored using deep learning to detect multiple lung diseases. For instance, Rana et al. [4] created an automated system for classifying 10 different lung diseases, utilizing a flexible CNN architecture in which graph neural networks were integrated with feedforward layers. A comprehensive analysis of deep learning applications in lung cancer diagnosis and classification is provided in a recent systematic review [5].

Object detection involves identifying and locating specific objects within an image, merging recognition with localization tasks. Through the application of modern object detection techniques, notable success has been achieved across various fields, including wildlife monitoring [6], autonomous driving [7], defect inspection [8], security surveillance [9], and face mask detection [10]. These advances have largely been driven by deep learning models, which are typically based on two architectures: convolutional neural networks (CNNs) [11,12,13,14,15,16] and self-attention-based Transformers [17]. CNN-based detectors are classified as either one-stage models, like the YOLO series [18,19,20], SSD [21], and RetinaNet [22], which prioritize speed, or two-stage models, such as the R-CNN family [23,24,25], which focus on accuracy. Recently, Transformer-based models like DETR [26] and Deformable DETR [27] have also gained popularity, reflecting ongoing innovations in the field of object detection.

Despite this progress, there are several challenges hindering the application of deep learning in medical image object detection [28,29,30]. For example, most deep learning object detection models are trained on the MS COCO dataset [31], which consists primarily of images unrelated to medical applications. This raises concerns about whether models trained on such datasets can perform well when applied to CXR images—a key issue this study seeks to address.

Another challenge is the costly and time-consuming process of labeling medical image data, especially for object detection tasks that require detailed annotations, such as adding bounding boxes around lesions. This task is even more difficult for CXR images, as it requires the expertise of radiologists, making the process more complex and resource-intensive [30]. As a result, the dataset used in this study contains only a limited number of training images for each disease category, with some categories having very few examples. This data scarcity poses a significant challenge for training deep learning models, for which large amounts of labeled data to avoid overfitting are required. To address this, we employ few-shot object detection methods, which are designed to recognize new (unseen) disease categories using only a few training examples after the model has been trained on numerous examples of known (seen) categories [32,33,34].

In this study, we aim to improve CXR image analysis by focusing on disease labels and location bounding boxes for object detection. We explore advanced deep-learning models, incorporating few-shot techniques to enhance their performance. Additionally, we compare various deep learning methods to evaluate their strengths and weaknesses, ultimately seeking to develop a more efficient and reliable system for the automated detection of chest diseases in CXR images.

## 2. Related Work

Substantial progress has been made in deep learning for image classification and object detection, impacting fields like medical imaging. However, accurately detecting specific disease markers in CXRs remains challenging, especially for rare conditions with limited data. A review of the current classification and detection methods reveals several gaps and limitations that serve as motivation for further research in this area.

### 2.1. Classification

Significant progress has been made in network architectures for image classification. Scaling up neural networks by increasing their depth can enhance accuracy but may also lead to the vanishing gradient problem. This was addressed through skip connections using ResNet [15] to improve gradient flow in deeper networks. DenseNet [16] is an expansion of ResNet with dense connections that allow each layer to receive feature maps from all previous layers, enabling feature reuse across layers to reduce parameter count and improve efficiency. Model scaling was further optimized in developing EfficientNet (B0–B7) [35] by balancing depth, width, and resolution, achieving a strong trade-off between accuracy and computational cost.

However, while these classification models are powerful for general tasks, they are limited in their ability to localize and classify the smaller more subtle abnormalities often found in CXRs, which require precise object detection capabilities beyond merely classification.

### 2.2. Object Detection

Different object detection architectures, typically categorized as one- or two-stage detectors, each have strengths and weaknesses. Two-stage detectors like those derived from the R-CNN framework are generally more accurate as they utilize a refined candidate selection process that filters out negative samples early, while one-stage detectors, exemplified by the YOLO series, focus on real-time detection but often generate too many candidate boxes, causing class imbalance by overfocusing on background samples. The focal loss function introduced in RetinaNet [22] improved the one-stage detector by reducing the influence of easy samples and enhancing learning from difficult samples. RetinaNet also uses a feature pyramid network [36] to integrate features from feature maps of different scales, thereby enhancing its feature extraction capability. YOLOv3 was improved using a decoupled head design, stronger data augmentation, and a shift to an anchor-free framework in developing the real-time detector YOLOX [37], with fewer parameters and better generalization. The problem of fixed IoU thresholds was addressed using Dynamic R-CNN [38] by dynamically adjusting the threshold during training and refining the loss function based on regression label statistics. DETR [26] revolutionized object detection by employing the Transformer architecture for end-to-end detection, eliminating the need for traditional anchor boxes or region proposals, though it suffers from slow convergence and poor small object detection. These issues were addressed using Deformable DETR [27], which focuses attention on key points near a reference point, improving performance for high-resolution images and small object detection.

Although these models have been adapted for various fields, their direct application to CXR analysis remains problematic due to issues such as high computational demand, slow convergence, and difficulties in detecting small but clinically relevant features. This highlights the need for more specialized object detection approaches that can overcome these limitations within the context of medical imaging.

### 2.3. Few-Shot Object Detection (FSOD)

The aim of few-shot learning is to build a model that can accurately classify images using very few training examples for specific classes. In FSOD, the categories are divided into base classes (with many training examples) and novel classes (with fewer examples). There are two stages in the training process: base training and k-shot fine-tuning. During base training, the model is only trained on base class objects, even if the images also contain novel class objects. In the k-shot fine-tuning stage, a small number (k) of bounding boxes from each class are used to refine the model. This approach is particularly useful in medical image analysis, where it may be difficult to collect data, with some diseases being extremely rare.

Meta-learning, which focuses on “learning to learn”, is crucial for FSOD, where models are trained on tasks from dataset subsets to rapidly adapt to new tasks. This fine-tuning approach was previously considered less effective until the two-stage fine-tuning approach TFA [39] challenged this view. TFA, built on Faster R-CNN, was initially trained on base classes and fine-tuned only the box predictor for all classes, improving accuracy by replacing the fully connected classifier with a cosine similarity-based classifier. The classification accuracy for novel classes was improved through FSCE [40] by using contrastive learning to separate novel instances from base classes. The contrastive proposals encoding loss were added to the Faster R-CNN loss, enhancing accuracy. In Meta-DETR [41], the first image-level few-shot detector, generalization was improved by incorporating the correlational aggregation module to capture inter-class correlations and reduce misclassification.

While these methods have shown success in domains like Pascal VOC and MS COCO, their performance on CXR datasets remains underexplored. Current FSOD techniques often struggle with inter-class variability and can suffer from misclassification, particularly in complex medical datasets where diseases may have overlapping visual features. Thus, developing a specialized FSOD approach for CXRs could significantly enhance model adaptability and reliability in detecting rare diseases.

### 2.4. Deep Learning-Based Object Detection for CXR Images

In recent years, deep learning-based object detection has been applied to CXR images for identifying foreign objects [42] and localizing abnormalities [43,44] in assisting the diagnosis of various diseases. Advanced architectures such as YOLO, RetinaNet, Mask R-CNN, and Faster R-CNN have been adapted for CXR analysis, achieving high accuracy and fast localization [45]. Notably, in a direct comparison of performance, the YOLOX model surpassed radiologists [44]. Large datasets with ground-truth bounding boxes, such as VinDr-CXR (open dataset of 18,000 CXRs with 28 abnormalities) [46] and CXR-AL14 (dataset available upon request for 165,988 CXRs with 14 abnormalities) [44], have been created to enhance model training.

To improve nodule detection performance, Behrendt et al. [47] evaluated strategies such as transfer learning using pre-trained weights from the VinDr-CXR and COCO datasets, as well as training from scratch. They addressed class imbalance by augmenting training data with generated nodules in healthy CXRs and compared this to oversampling the less frequent class (CXRs with nodules). After testing various state-of-the-art object detection algorithms, they developed a systematic approach that incorporated the most effective techniques, ultimately outperforming all competitors in the NODE21 competition’s detection track [48].

## 3. Materials

### 3.1. Dataset

We used a dataset containing 2123 CXR images, featuring both normal cases and 18 types of diseases. The images were in the DICOM format. These images were retrospectively collected from the archiving and communication system (PACS) at E-Da Hospital, covering patient CXRs from January 2008 to December 2018. Along with the images, the dataset included patient information such as gender, age, and diagnostic reports from radiologists. The Institutional Review Board of E-Da Hospital approved this study, and all patients provided written informed consent.

The 18 disease types were chosen by reviewing diagnostic reports and selecting those with reliable high-quality annotations. We also prioritized diseases with visual manifestations that could be effectively detected by object detection algorithms, making them suitable for automated analysis.

An experienced radiological physician (K.-Y.L.) identified and marked lesion regions on the image. The rectangular bounding box was carefully placed to closely surround the lesion, capturing its full extent while minimizing any inclusion of unaffected tissue. After this initial placement, another senior radiologist (N.-H.L.) reviewed and confirmed that bounding boxes were accurately sized and precisely positioned.

Images were excluded if they were of poor quality or had unclear diagnostic reports. We also excluded images of minors (patients under 18). After removing duplicates and missing data, we retained 1802 images, each representing a unique patient. The image sizes varied, with heights ranging from 1304 to 4280 pixels and widths from 1066 to 4280 pixels. The dataset employs multi-label classification, as a single patient can have multiple diseases. Considering the number of cases as well as the sizes and locations of bounding boxes, we grouped the 18 diseases into 12 categories based on medical guidance. Table 1 presents the number of images before and after merging the diseases, with the abbreviations of the 12 disease names provided for simplicity. Notably, the number of normal cases is much higher than the combined total of the 12 diseases, indicating there is a significant class imbalance in the dataset.

**Figure 1:**
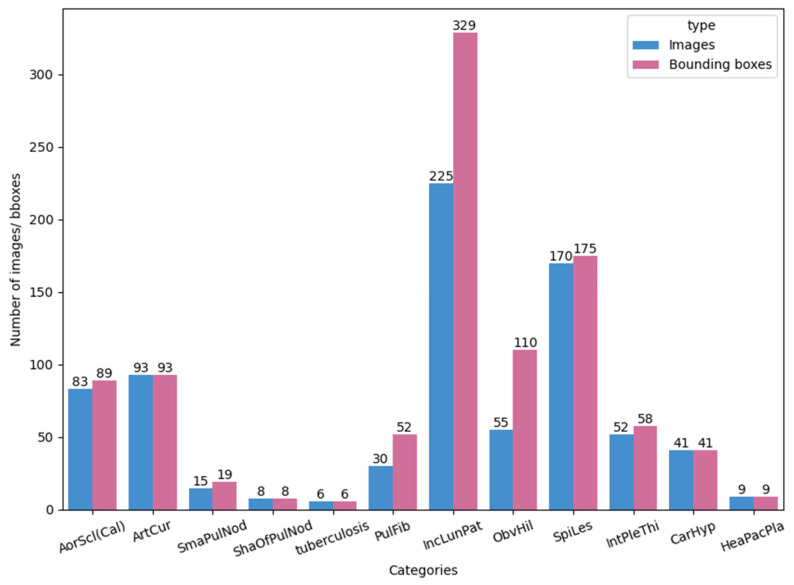
The number of images and bounding boxes for each of the 12 disease categories.

**Figure 2:**
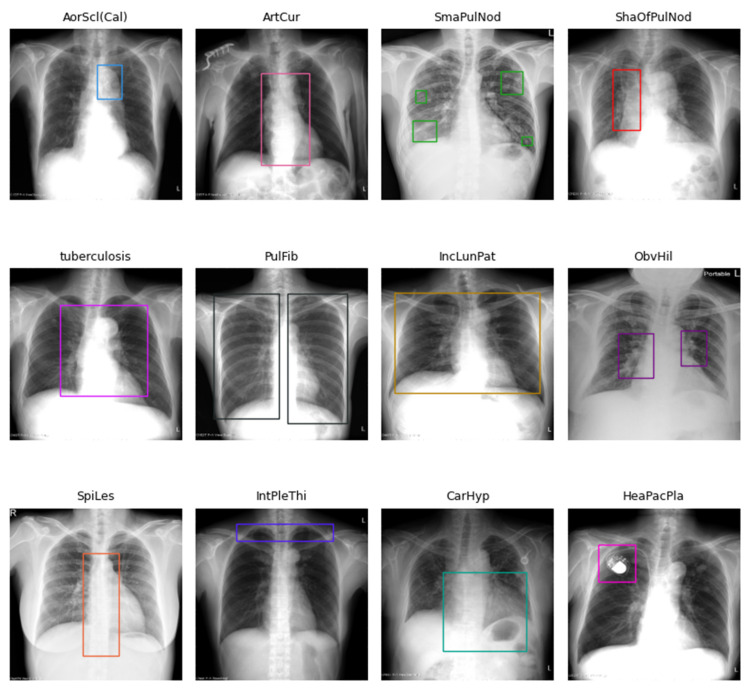
Examples of X-ray images and their corresponding bounding boxes across the 12 disease categories.

Each image in the 12 disease categories contains one or more bounding boxes. For example, annotations for ObvHil (obvious hilar) and PulFib (pulmonary fibrosis) are often paired, while multiple bounding boxes are typical for SmaPulNod (small pulmonary nodules). Figure 1 is a bar chart illustrating the total number of images and bounding boxes for each disease category, and Figure 2 shows sample X-ray images with their corresponding bounding boxes across the 12 categories.

### 3.2. Data Preprocessing

We processed the CXR images using header data embedded in the DICOM files. If the DICOM file indicated a logarithmic relationship between pixel values and X-ray beam intensity, we applied “intensity log transformation”. In this process, each pixel value x[i] is adjusted based on the visible range defined by the Window Center (WC) and Window Width (WW). The visible pixel range is between $iMin=WC-\frac{WW}{2}$ and $iMax=WC+\frac{WW}{2}$, while the number of bits for each pixel is defined by BitsStored. The steps for the intensity log transformation are depicted in Algorithm 1.
**Algorithm 1** Pseudocode of the intensity log transformationInput: x**  for** i=0,⋯,N−1 **do****   if** x[i]<iMin, **then** x[i]=iMin   **if** x[i]>iMax, **then** x[i]=iMax$\;\;\;z\left[i\right]=-\log\left(\frac{1+x\left[i\right]}{2^{BitsStored}}\right)$  
**end for**
Output: z

**Figure 3:**
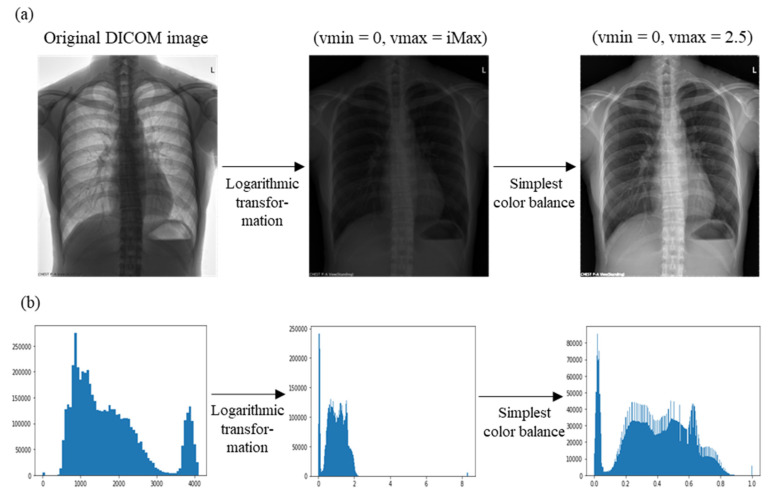
(**a**) X-ray images before and after data preprocessing and (**b**) their corresponding intensity histograms.

CXR images often contain elements, such as chest markers, that are irrelevant to disease detection. These markers often appear overexposed after logarithmic transformation, such as in the example of letter “L” (Figure 3a). To enhance the areas of interest, we adjusted image contrast using the “simplest color balance algorithm”, in which saturation limits of $v_{min}$ and $v_{max}$ are set to improve contrast (Algorithm 2).
**Algorithm 2** Pseudocode of the simplest color balanceInput: z  **for** i=0,⋯,N−1 **do**$\;\;\;c\left[i\right]=\frac{z\left[i\right]-v_{min}}{v_{max}-v_{min}}$   **if** $c\left[i\right]<0$, **then** c[i]=0   **if** $c\left[i\right]>1$, **then** c[i]=1  
**end for**
Output: c

In this study, we set $v_{min}=0$ and $v_{max}=2.5$. Figure 3 shows the progression from the original DICOM image, through intensity log transformation, to the final contrast-adjusted image using the simplest color balance algorithm. Intensity histograms for each step are also shown.

### 3.3. Experimental Data Setups

To assess the impact of having a large proportion of normal images in our dataset, a common issue in clinical practice, we created three datasets for our object detection models. First, the entire dataset (1802 samples), labeled Dataset A, was divided into training, validation, and test sets with approximate proportions of 63.4%, 16.5%, and 20.1%, respectively, while ensuring similar disease distributions across all subsets. Next, we created Dataset B by removing two-thirds of the normal images from the training set and Dataset C by removing all normal images from the training set. Table 2 shows the number of images in each dataset.

For the FSOD models, we performed an extra step, dividing the disease categories into base and novel classes for base training and k-shot fine-tuning. We used the same three datasets created earlier, designating categories with fewer images—SmaPulNod, ShaOfPulNod (shadows of pulmonary nodules), tuberculosis, PulFib, and HeaPacPla (heart pacemaker placement)—as novel classes. The remaining seven categories were treated as base classes. During k-shot fine-tuning, we set k to 1, 2, 3, 5, or 10, meaning that we randomly selected up to 10 images for annotation per novel category. When there were fewer than 10 images in a category, we used all of the available images. An image selected for one category was not reused for another.

## 4. Methods

In this study, we applied object detection and FSOD methods to identify disease types and lesion areas in CXR images. We designed four analytical schemes to determine the most effective approach. These schemes involved using either object detection or the FSOD models, with or without a preliminary binary classification step to determine the presence of disease in the image. For binary classification, object detection, and FSOD tasks, we selected two, five, and three models, respectively, as shown in Table 3. To understand the impact of model architecture on performance for both object detection and FSOD tasks, we chose models from two primary categories: CNN-based and Transformer-based models. Additionally, we calculated the specificity of normal images in the test set for each scheme to assess whether the models could maintain a low misdiagnosis rate while excelling at disease detection.

### 4.1. Scheme 1: Object Detection

**Figure 4:**
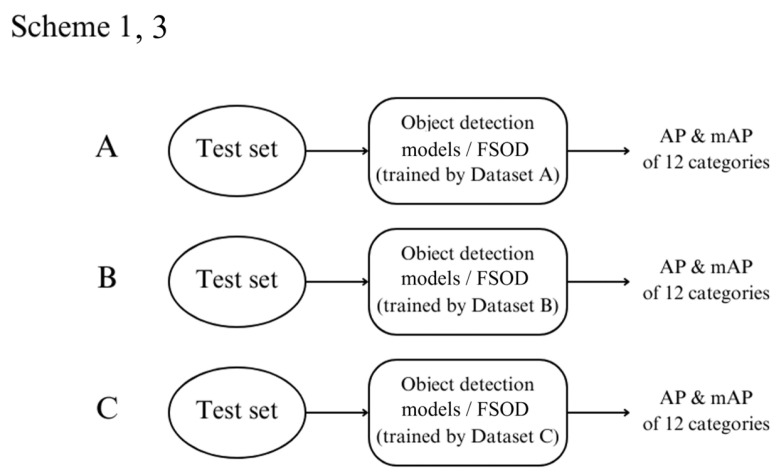
Flowchart for Schemes 1 and 3, outlining the steps involved in object detection or few-shot object detection (FSOD).

In Scheme 1, we trained object detection models using three datasets: A, B, and C. After training, we tested these models on the test set and calculated key evaluation metrics, including average precision (AP) and mean average precision (mAP), for detecting diseases in the 12 disease categories. The overall process is depicted in Figure 4.

### 4.2. Scheme 2: Binary Classification + Object Detection

In this scheme, a binary classification step is introduced before object detection. Two classification models, Classification Models A and B, were trained on Datasets A and B, respectively, to determine whether a patient had any disease. Classification Model A uses the EfficientNet-B3 architecture, while Classification Model B employs DenseNet121.

During testing, images from the test set were first classified by the binary models. Images classified as positive (indicating the presence of disease) were passed onto the object detection models trained in Scheme 1. Those classified as negative (indicating no disease) were labeled as normal and were not subjected to further object detection.

**Figure 5:**
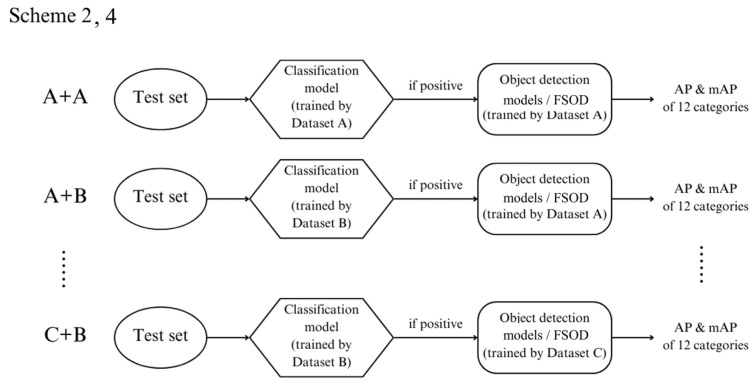
Flowchart for Schemes 2 and 4, illustrating the process for binary classification followed by object detection or few-shot object detection (FSOD).

Since Scheme 1 involves training object detection models on three datasets and this schedule includes classification models on two datasets; there are six possible outcomes for each test image: A + A, A + B, B + A, B + B, C + A, and C + B. For instance, in the A + B case, the image was first classified by the classification model trained on Dataset B and, if positive, analyzed by the object detection model trained on Dataset A. Figure 5 illustrates this process.

### 4.3. Scheme 3: Few-Shot Object Detection

Scheme 3 mirrors Scheme 1 but is focused on the FSOD models. These models were trained on three datasets (A, B, and C), and AP and mAP were calculated for detecting the 12 diseases using the test set.

### 4.4. Scheme 4: Binary Classification + Few-Shot Object Detection

This scheme is similar to Scheme 2 except that after binary classification, the FSOD models from Scheme 3 are used for further detection. Like Scheme 2, this method generates six possible outcomes: A + A, A + B, B + A, B + B, C + A, and C + B.

### 4.5. Evaluation Metrics

To assess the performance of the binary classifiers, we used standard metrics: accuracy, precision, recall, and F1-score. For object detection and image segmentation, we used the intersection over union (IoU) metric, which measures the overlap between a predicted bounding box (Pred) and the ground-truth box (GT). The IoU is calculated as follows:

$$IoU=\frac{\left|GT\cap Pred\right|}{\left|GT\cup Pred\right|},\;\;\;0\leq IoU\leq 1$$

Here, $\left|GT\cap Pred\right|$ represents the overlapping pixels between the predicted and ground truth boxes, and $\left|GT\cup Pred\right|$ is the total number of pixels in both boxes. An IoU of 0 indicates no overlap, while an IoU of 1 indicates a perfect match. We set a threshold of 0.5 for this study, meaning that predictions with IoU values above this threshold are considered correct.

In object detection, the mean average precision (mAP) is a key metric for evaluating model performance. It combines precision and recall by calculating the average precision (AP) for each class. For M object classes, the AP for the mth class is calculated as follows:

$${AP}_{m}=\int_{0}^{1}{PR}_{m}\left(r\right)dr$$

where ${PR}_{m}\left(r\right)$ is the precision–recall curve for the mth class. To compute precision and recall, predicted boxes are ranked based on confidence scores. If the IoU between a predicted and ground–truth box exceeds the threshold, it is considered a true positive; otherwise, it is a false positive. After calculating precision and recall for all predictions, the precision–recall curve is plotted, and the area under the curve is calculated for each class. The mAP is then calculated as the average of APs across all classes:

$$mAP=\frac{1}{M}\sum_{m=1}^{M}{AP}_{m}$$

The mAP score ranges from 0 to 1, with values closer to 1 indicating better model performance in detecting and localizing objects.

## 5. Results

We present the results from experiments conducted on three custom-designed datasets using four different analysis approaches. Two binary classification models were trained on a P100 GPU, while the object detection and FSOD models were trained using NVIDIA GeForce RTX 3080 and GTX 1080 Ti GPUs.

### 5.1. Binary Classification

EfficientNet-B3 was trained on Dataset A, and DenseNet121 on Dataset B. These models were used in Schemes 2 and 4 to predict whether an image contained at least one instance of disease. Pretrained ImageNet weights were used, with only the fully connected layer retrained. The hyperparameters are listed in Supplementary Table S1, and images were normalized using the ImageNet mean and standard deviation.

Table 4 presents the performance of these models on the test set. Due to class imbalance, we used both the accuracy and F1-score for evaluation. EfficientNet-B3, trained on Dataset A, outperformed DenseNet121, trained on Dataset B, across all metrics except precision.

### 5.2. Comparison of Analytic Schemes

The training hyperparameters for object detection and FSOD models are provided in Supplementary Tables S2 and S3.

#### 5.2.1. Results on mAP

**Figure 6:**
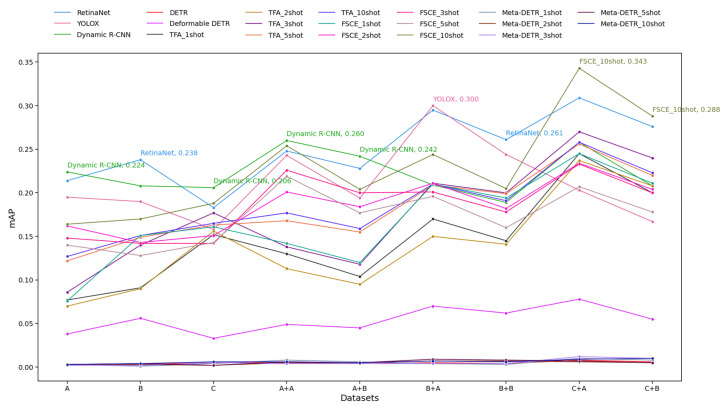
Mean average precision (mAP) results for Schemes 1 to 4. The y-axis represents mAP values, while the x-axis shows the datasets used for model training. “A” refers to training the object detection model on Dataset A (Schemes 1 and 3), while “A + B” indicates first using the classification model trained on Dataset B and then the object detection model trained on Dataset A (Schemes 2 and 4). The same applies to other labels. For the object detection models, each line is the performance of the indicated model on the test set. For the FSOD models, the five lines are the performance for each few-shot fine-tuning scenario (1-shot, 2-shot, 3-shot, 5-shot, 10-shot). The mAP of the best-performing model is shown for each dataset labeled on the *x*-axis.

Figure 6 shows the mAP performance of various object detection models for Schemes 1 to 4 across multiple datasets. Here, AP was calculated using IoU greater than 0.5 (mAP@0.5).

Some key patterns are observed. 1. Overall Performance: Processing test images through a binary classification model before object detection yielded better results. 2. Top Performers: FSCE 10-shot consistently achieved top mAP values, particularly on the C + A dataset, where it peaked at 0.343. YOLOX also performed well, peaking at 0.300 on the B + A dataset, although it did not maintain top performance across all datasets. Dynamic R-CNN and RetinaNet also performed competitively, with notable peaks around 0.26 and 0.261, respectively. 3. Low Performers: The three Transformer-based models consistently achieved very low mAP values across datasets, with DETR and Meta-DETR maintaining a flat trend near zero. 4. Few-Shot Trends: Models trained with a higher number of shots generally performed better, with the TFA and FSCE 10-shot achieving higher mAP than TFA and FSCE 1-shot across datasets. However, the mAP gains from increasing the shot number were not always linear and varied across datasets. 5. Dataset Influence: Training classification models on Dataset B appeared to be more challenging, as evidenced by the lower performance of models when trained on the x + B rather than the x + A dataset. Performance generally peaked on the C + A dataset, where more models achieved their highest mAP values. 6. Model Stability: Some models, such as Dynamic R-CNN and RetinaNet, exhibited greater stability with relatively smaller fluctuations in mAP across datasets. On the other hand, YOLOX and FSCE had more variability, suggesting that their performance may be more sensitive to dataset characteristics. In summary, Figure 6 shows that processing test images with a binary classification model before object detection generally improved results, with FSCE 10-shot achieving the highest mAP values, especially on the C + A dataset. Models like YOLOX, Dynamic R-CNN, and RetinaNet performed well but showed varying stability across datasets, while Transformer-based models (DETR, Meta-DETR) consistently had low mAP values.

#### 5.2.2. Results for Base and Novel mAP

In FSOD, the categories were divided into base and novel classes. During base training, only base class bounding boxes were used, with novel classes reserved for few-shot fine-tuning. It was expected that the FSOD models would perform better on novel classes, on account of their fewer samples, while the traditional object detection models would excel on base classes.

**Figure 7:**
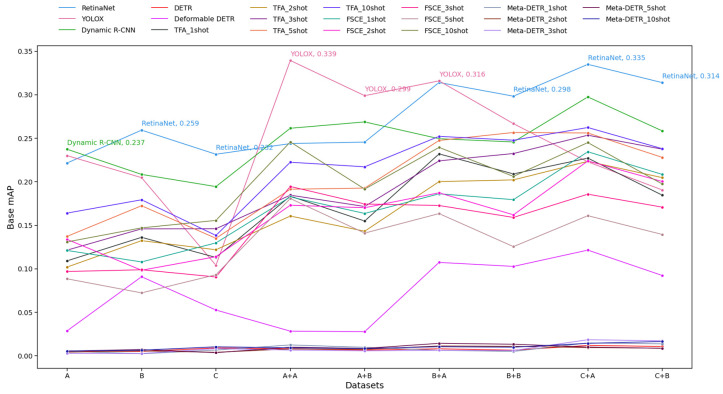
Base mean average precision (base mAP) results for Schemes 1 to 4. The y-axis represents base mAP values, while the x-axis shows the datasets used for model training. “A” refers to training the object detection model on Dataset A (Schemes 1 and 3), while “A + B” indicates first using the classification model trained on Dataset B and then the object detection model trained on Dataset A (Schemes 2 and 4). The same applies to other labels. For the object detection models, each line is the performance of the indicated model on the test set. For the FSOD models, the five lines are the performance for each few-shot fine-tuning scenario (1-shot, 2-shot, 3-shot, 5-shot, 10-shot). The base mAP of the best-performing model is shown for each dataset labeled on the *x*-axis.

**Figure 8:**
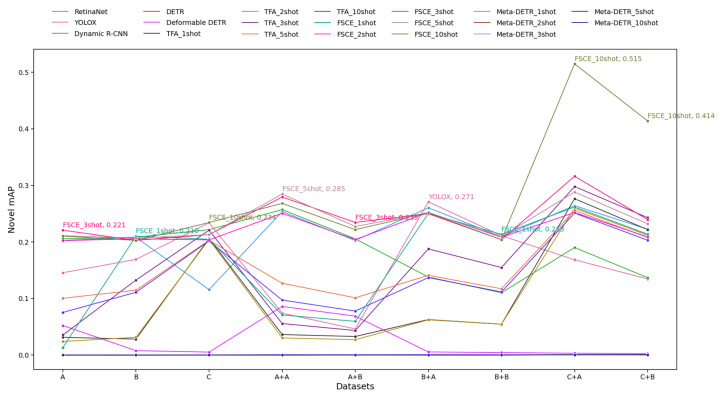
Novel mean average precision (novel mAP) results for Schemes 1 to 4. The y-axis represents novel mAP values, while the x-axis shows the datasets used for model training. “A” refers to training the object detection model on Dataset A (Schemes 1 and 3), while “A + B” indicates first using the classification model trained on Dataset B and then the object detection model trained on Dataset A (Schemes 2 and 4). The same applies to other labels. For the object detection models, each line is the performance of the indicated model on the test set. For the FSOD models, the five lines are the performance for each few-shot fine-tuning scenario (1-shot, 2-shot, 3-shot, 5-shot, 10-shot). The novel mAP of the best-performing model is shown for each dataset labeled on the *x*-axis.

Figure 7 and Figure 8 show the mAP for base and novel classes of various object detection models across a series of datasets. First, we discuss base classes. 1. Top Performers: YOLOX achieved the highest base mAP value of 0.339 on the A + A dataset. It consistently ranked among the top-performing models across multiple datasets. RetinaNet also performed strongly, with a peak of 0.335 and high base mAP values across several datasets. It exhibited more consistent performance with smaller fluctuations than YOLOX. Dynamic R-CNN also performed relatively well, although its base mAP values were slightly lower and showed some variability compared to RetinaNet. 2. Low Performers: The three Transformer-based models consistently had very low base mAP values across datasets, with DETR and Meta-DETR remaining almost flat near zero. 3. Few-Shot-Based Model Performance: Higher-shot FSOD models (such as FSCE 10-shot) tended to have better base mAP values than their lower-shot counterparts but were generally outperformed by traditional object detection models such as YOLOX and RetinaNet on most datasets. 4. Model Stability: RetinaNet and Dynamic R-CNN showed trends of more stable performance, with fewer abrupt changes in base mAP across datasets. YOLOX, while generally performing well, had some larger fluctuations in base mAP, indicating that it may be more sensitive to changes in dataset characteristics. Overall, Figure 7 highlights that YOLOX and RetinaNet performed well and were relatively robust across datasets, while DETR and Meta-DETR consistently underperformed. Models trained with a higher number of shots (e.g., FSCE 10-shot) generally achieved better mAP than lower-shot variants, though not at the level of the top models like YOLOX and RetinaNet.

Second, we discuss novel classes. 1. Top Performers: FSCE 10-shot was the best-performing model, reaching a peak novel mAP of 0.515 on the C + A dataset and another high of 0.414 on the C + B dataset. This suggests that FSCE 10-shot was particularly effective at handling novel data. Other FSCE variants and YOLOX also performed well, achieving top novel mAP values across multiple datasets. 2. Low Performers: DETR and Meta-DETR continued to exhibit low performance, with novel mAP values near zero across most datasets. The traditional object detection models, such as Dynamic R-CNN and RetinaNet, showed relatively low novel mAP values, suggesting they may not generalize as well to novel classes. 3. Few-Shot Trends: Models trained with a higher number of shots (e.g., FSCE 5-shot and FSCE 10-shot) generally performed better on novel classes than their lower-shot counterparts. The increase in mAP with higher shot numbers suggests that these models benefited from having additional samples to learn novel object detection. 4. Dataset Influence: Training classification models on Dataset B appeared to be more challenging, given the lower performance of models trained on the x + B than on the x + A dataset, and there was a general peak in performance on the C + A dataset, with more models achieving their highest mAP values. In summary, Figure 8 highlights that FSCE (particularly 10-shot) excelled in novel detection tasks, and YOLOX also performed well. Higher-shot models generally performed better in detecting novel objects, while lower-shot and non-few-shot models struggled, particularly on challenging datasets.

#### 5.2.3. Disease-Wise AP Results

We evaluated model performance for each disease category, as shown in Supplementary Figures S1–S4. For the FSOD models, only the 10-shot fine-tuning results are presented. In Schemes 1 and 2, DETR performed poorly, detecting a few lesions in the categories ArtCur (arterial curvature) and SpiLes (spinal lesions). Similarly, Meta-DETR, a Transformer-based model, underperformed in Schemes 3 and 4, showing only limited lesion detection in the IncLunPat (increased lung patterns) category. By contrast, the CNN-based models performed well in most categories, with object detection models excelling in the categories IncLunPat, SpiLes, CarHyp (cardiac hypertrophy), and HeaPacPla. The FSOD models performed better when the test images were first processed through a classification model. The best prediction results were observed for HeaPacPla, with many models demonstrating strong performance. For the lower-performing category ShaOfPulNod, its AP can be boosted to as high as 1 using FSCE with the combinations C + A or C + B.

We also investigated whether the number of shots used in fine-tuning affects novel class performance. Supplementary Figure S5 shows the results of FSCE, the best-performing FSOD model, for five novel classes: SmaPulNod, ShaOfPulNod, tuberculosis, PulFib, and HeaPacPla. For SmaPulNod and ShaOfPulNod, models were trained on Dataset C with 10-shot fine-tuning outperforming the others. For PulFib, better performance was achieved using 1-shot and 5-shot fine-tuning, while for tuberculosis, Classification Model A misclassified images as normal, preventing AP calculation for the combinations A + A, B + A, and C + A. HeaPacPla achieved perfect predictions with 2- or higher-shot fine-tuning.

#### 5.2.4. Accuracy of Normal Images

**Figure 9:**
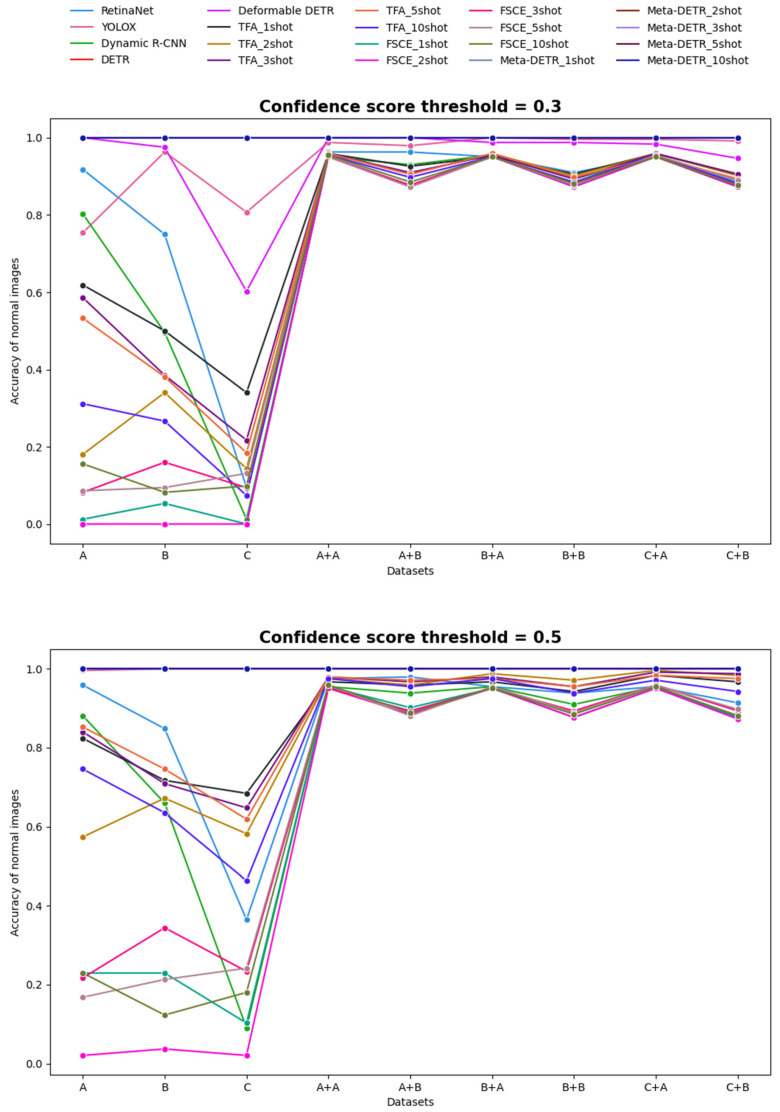
Accuracies of normal images for Schemes 1 to 4. The top graph shows the results using a confidence score threshold of 0.3, while the bottom graph displays the results for a threshold of 0.5.

To avoid misclassifying normal images as diseased, we calculated the specificity (accuracy of normal images) across the four schemes using confidence score thresholds of 0.3 and 0.5. If a predicted bounding box on a normal image exceeded the threshold, the image was considered misclassified. Figure 9 shows the specificities across all methods. Models that passed test images through a classification model before object detection achieved significantly higher specificity. The three Transformer-based models, despite their lower mAP performance indicated earlier, showed near-perfect specificity. Transformer-based models often excel in capturing the global context due to their self-attention mechanism, which enhances their ability to differentiate between object and non-object regions and thereby more accurately classify normal images, contributing to their higher specificity. However, they struggle with the localization and accurate detection of fine-grained objects and may require more extensive training data or context diversity to achieve high precision and recall, which explains the discrepancy between their strong specificity and weaker mAP. In contrast, models like Dynamic R-CNN, TFA, and FSCE (all based on the faster R-CNN framework), which had higher mAP, tended to show less satisfactory specificity. This may be due to the design and training objectives of the faster R-CNN architecture, where their region proposal networks excel at generating candidate regions to contain objects, but are more prone to misclassify background or non-object regions as objects when confident regions are identified. Thus, these CNN-based models are tuned to prioritize sensitivity in finding objects, potentially sacrificing specificity. The specificity of the two FSOD models, TFA and FSCE, decreased as the number of shots used in fine-tuning increased. YOLOX stood out by achieving almost perfect specificity at the 0.5 threshold, excelling in both mAP and the accuracy of normal image detection.

## 6. Discussion

In this study, we developed deep learning-based object detection strategies with two main goals: first, to address the class imbalance in the dataset for more accurate predictions, and second, to reduce the false positive rate while maintaining high accuracy. To achieve these objectives, we established several datasets and experimental approaches. The results showed that the CNN-based models consistently outperformed the Transformer-based models, and the proportion of background images in the training sets had a significant effect on the inference capabilities of these models. When comparing the four proposed analytic schemes, we found that Schemes 2 and 4, which first applied a classification model, outperformed Schemes 1 and 3, which relied solely on object detection or the FSOD models. In particular, the best results were obtained using the approach where test images were first processed by Classification Model A and then by FSCE trained on Dataset C with 10-shot fine-tuning.

Despite attempts to balance the data by adjusting the proportion of background images in the training datasets, class imbalance remained an issue. This led to the use of FSOD models in Schemes 3 and 4, which were expected to handle class imbalance better based on their architecture. The experimental results confirmed that the FSOD models outperformed the object detection models for novel classes, while the reverse was true for base classes. Class-wise AP analysis showed that different k-shot fine-tuning settings affected categories in varying ways; more shots did not always result in better performance.

To achieve our second goal, we also calculated the specificity of the test data (i.e., accuracy for normal image detection) across all four schemes. The results indicated that the accuracy of normal images could first be improved by using a binary classification model. Overall, models that excelled in terms of mAP tended to have lower accuracy for normal images, and vice versa. YOLOX was the only model that performed well in terms of both mAP and normal image accuracy.

Studies have shown that CNN architectures are particularly effective in detecting and localizing abnormalities in CXR images [45]. In our study, we also found that CNN-based models, particularly YOLOX and FSCE with 10-shot fine-tuning, achieved the highest mAP scores in disease detection. The limitations posed by small imbalanced annotated datasets in developing deep learning models for localization have been highlighted in previous research and addressed by using transfer learning and augmentation techniques [47], in combination with large datasets containing ground-truth bounding boxes [44]. Similarly, we observed that FSOD techniques, like FSCE, significantly enhanced the accuracy in detecting diseases with limited samples. The use of Transformer-based object detection models in medical image analysis is less common. In our study, we extended the literature by showing that, unlike CNNs, Transformer-based models such as DETR and Meta-DETR, which excel in general object detection tasks on diverse datasets such as COCO, exhibited lower performance in CXR disease detection. While most studies have focused on a single detection model for specific tasks, Behrendt et al. [47] distinguished themselves by evaluating transfer learning, nodule augmentation, and various detection algorithms in building a robust nodule detection system. Our study further contributes by systematically comparing CNN- and Transformer-based object detection algorithms along with FSOD techniques to identify and fine-tune the most suitable deep learning models for various disease detection tasks in CXR images.

The proposed methods for disease detection and localization in CXRs show significant promise, yet there are limitations that affect their robustness and scalability in clinical settings. First, the models relied on a single-institution dataset, which may lead to overfitting and reduce their generalizability to diverse clinical environments with varying imaging protocols and patient populations. Although FSOD techniques were employed to address class imbalance, accurate detection remained a challenge for certain categories of rare diseases with very few examples. Transformer-based models like DETR and Meta-DETR also exhibited limitations in detecting small abnormalities in CXR images, and their high computational demands further limit their feasibility in real-time or resource-constrained settings. Additionally, the reliance on precise bounding box annotations introduces potential subjectivity, impacting localization accuracy. Future research could focus on enhancing model generalizability by incorporating multi-institutional datasets and reducing class imbalance through data augmentation. Exploring lightweight model architectures or hybrid approaches that integrate CNNs with Transformers could allow for optimizing performance while reducing computational requirements. Improved annotation techniques, such as weak- or self-supervised learning [34] or semi-automated labeling [49,50] may also be used to enhance model training quality and overall detection accuracy, paving the way for more robust and clinically viable AI-based diagnostic tools.

## 7. Conclusions

This study explored the application of deep learning-based object detection models for disease detection and localization in CXR images. By employing CNN and Transformer-based architectures, as well as FSOD techniques, we developed and evaluated approaches for accurately detecting 12 thoracic diseases across multiple analytic schemes. Our results indicate that the CNN-based models, particularly YOLOX and FSCE (10-shot), consistently achieved high mAP scores, underscoring their robustness and adaptability to clinical settings. In comparison, Transformer-based models such as DETR and Meta-DETR exhibited limitations in small object localization, which may stem from both the model architecture and dataset constraints. Our approach highlights the potential of binary classification as a preliminary step to reduce false positives in object detection, leading to improved disease detection accuracy and specificity for normal images. Furthermore, incorporating FSOD enhanced the capability of our model to handle rare diseases with minimal training samples, suggesting that few-shot learning can be a valuable addition to resource-constrained medical imaging tasks.

While promising results were achieved, certain limitations, including potential overfitting to dataset-specific features, high computational demands, and class imbalances, highlight avenues for future research. These limitations could be addressed through approaches such as cross-institutional validation, lightweight model design, and data augmentation to further enhance model precision and clinical applicability. Ultimately, this work contributes valuable insights to the development of robust automated diagnostic tools, paving the way toward more accurate and efficient CXR disease detection in real-world healthcare settings.

## Figures and Tables

**Table 1:** Number of images in each disease category before and after merging.

Before		After		
Categories	Count	New Categories	Abbr.	Count
Normal	1212	Normal	Normal	1212
Aortic arch atherosclerotic plaque	28	Aortic sclerosis (calcification)	AorScl(Cal)	83
Aortic arch calcification	16			
Aortic atherosclerosis	25			
Aortic wall calcification	20			
Aortic curvature	65	Arterial curvature	ArtCur	93
Thoracic vertebral artery curvature	28			
Small pulmonary nodules	15	Small pulmonary nodules	SmaPulNod	15
Shadows of pulmonary nodules	8	Shadows of pulmonary nodules	ShaOfPulNod	8
Tuberculosis	6	Tuberculosis	tuberculosis	6
Pulmonary fibrosis	30	Pulmonary fibrosis	PulFib	30
Increased lung streak	89	Increased lung patterns	IncLunPat	225
Lung field infiltration	138			
Obvious hilar	55	Obvious hilar	ObvHil	55
Degenerative joint disease of the thoracic spine	75	Spinal lesions	SpiLes	170
Scoliosis	100			
Intercostal pleural thickening	52	Intercostal pleural thickening	IntPleThi	52
Cardiac hypertrophy	41	Cardiac hypertrophy	CarHyp	41
Heart pacemaker placement	9	Heart pacemaker placement	HeaPacPla	9

**Table 2:** Number of images in the three datasets used for object detection.

Categories ^1^	Training ^2^	Validation	Test	Total ^2^
Normal	779/178/0	189	244	1212/611/433
AorScl(Cal)	52	15	16	83
ArtCur	57	18	18	93
**SmaPulNod**	9	3	3	15
**ShaOfPulNod**	6	1	1	8
**tuberculosis**	3	1	2	6
**PulFib**	20	6	4	30
IncLunPat	130	42	53	225
ObvHil	34	12	9	55
SpiLes	107	28	35	170
IntPleThi	35	8	9	52
CarHyp	23	9	9	41
**HeaPacPla**	6	1	2	9
Unique images	1143/542/364	297	362	1802/1201/1023

^1^ Categories in bold represent novel classes. ^2^ Datasets A, B, and C have the same number of images for each disease category but differ regarding the number of images for the normal category in the training set. For simplicity, the notation A/B/C is used to represent the number of images in Datasets A, B, and C, respectively.

**Table 3:** Models used in this study.

		Architecture	
		CNN-Based	Transformer-Based
Task	Binary classification	EfficientNet-B3 [35], DenseNet121 [16]	
	Object detection	RetinaNet [22], YOLOX [37], Dynamic R-CNN [38]	DETR [26], Deformable DETR [27]
	Few-shot object detection	TFA [39], FSCE [40]	Meta-DETR [41]

**Table 4:** Performance comparison of the two binary classification models.

	Accuracy	F1-Score	Precision	Recall
EfficientNet-B3 on Dataset A	88.12%	85.85%	84.41%	88.05%
DenseNet121 on Dataset B	86.74%	85.34%	86.44%	84.56%

## Data Availability

The data used and analyzed in this study are available from the corresponding author upon reasonable request.

## Supplementary Materials

The following supporting information can be downloaded at: https://www.mdpi.com/article/10.3390/diagnostics14232636/s1, Table S1. Hyperparameter settings for the binary classification models. Table S2. Hyperparameter settings for the object detection models. Table S3. Hyperparameter settings for the FSOD models. Figure S1. Disease-wise average precision (AP) values for Scheme 1. Figure S2. Disease-wise average precision (AP) values for Scheme 2. Figure S3. Disease-wise average precision (AP) values for Scheme 3. Figure S4. Disease-wise average precision (AP) values for Scheme 4. Figure S5. Novel class APs of FSCE for different shot settings.
