# Supplementary material for: Deep Learning-Based Object Detection Strategies for Disease Detection and Localization in Chest X-Ray Images
Source: Diagnostics (Basel). 2024 Nov 22;14(23):2636. doi: 10.3390/diagnostics14232636 (PMC11640298; doi:10.3390/diagnostics14232636)
Supplement: Supplementary file 1 [file diagnostics-14-02636-s001.zip › diagnostics-3277921-supplementary.pdf]

Deep Learning-Based Object Detection Strategies for Disease Detection and Localization in Chest X-Ray Images—Supplementary Material

Yi-Ching Cheng, Yi-Chieh Hung, Guan-Hua Huang, Tai-Been Chen, Nan-Han Lu, Kuo-Ying Liu and Kuo-Hsuan Lin

Table S1. Hyperparameter settings for the binary classification models.

	**EfficientNet-B3 (Classification Model A)**	**DenseNet121 (Classification Model B)**
**Epoch**	100	100
**Batch size**	16	16
**Learning rate**	0.0001	0.0001
**Weight decay**	0.001	0.001
**Data augmentation**	Resize(512, 512)<br>RandomHorizontalFlip<br>RandomBrightnessContrast<br>RandomColorJitter<br>Normalize	Resize(512, 512)<br>RandomHorizontalFlip<br>RandomBrightnessContrast<br>RandomColorJitter<br>Normalize
**Loss function**	Cross entropy	Cross entropy
**Optimizer**	Adam	Adam
**Scheduler**	OneCycleLR	OneCycleLR

Table S2. Hyperparameter settings for the object detection models.

	RetinaNet	YOLOX	Dynamic R-CNN	DETR	Deformable DETR
**Backbone** [1]	ResNet50	DarkNet53	ResNet50	ResNet50	ResNet50
**Epoch**	100	100	100	500	10
**Batch size**	4	4	4	2	2
**Learning rate**	0.0002	0.0002	0.0002	0.00002	0.00002
**Momentum**	0.9	0.9	0.9		
**Weight decay**	0.0001	0.0005	0.0001	0.0001	0.0001
**Data augmentation**	RandomChoiceResize[(1333, 640), (1333, 800)], RandomHorizontalFlip, Normalize	Resize(640, 640), Mosaic, MixUp, RandomAffine, Normalize, RandomHorizontalFlip	Resize(1333, 800), RandomHorizontalFlip, Normalize	RandomChoice[RandomChoiceResize(x, 1333) [2], RandomCrop], RandomHorizontalFlip, Normalize	RandomChoice[RandomChoiceResize(x, 1333) [2], RandomCrop], RandomHorizontalFlip, Normalize
**Loss function**	L1 loss (reg), focal loss (cls)	IoU loss (reg), cross entropy (cls, obj)	head: SmoothL1 loss (reg), cross entropy (cls), RPN: L1 loss (reg), cross entropy (cls)	L1 loss (reg), cross entropy (cls), GIoU loss (bbox)	L1 loss (reg), focal loss (cls), GIoU loss (bbox)
**Optimizer**	SGD	SGD	SGD	AdamW	AdamW
**Scheduler**	MultiStepLR	CosineAnnealingLR, ConstantLR	MultiStepLR	MultiStepLR	MultiStepLR

[1] Backbones were pretrained on ImageNet.

[2] Here, x refers to the following scales: [(400, 500, 600), (480, 512, 544, 576, 608, 640, 672, 704, 736, 768, 800)].

Table S3. Hyperparameter settings for the FSOD models.

	TFA	FSCE	Meta-DETR
**Backbone** [1]	ResNet101	ResNet101	ResNet50
**Epoch**	100 (base training), 800 (few-shot fine-tuning)	100 (base training), 800 (few-shot fine-tuning)	30 (base training), 500 (few-shot fine-tuning)
**Batch size**	2	2	1
**Learning rate**	0.0025	0.0025	0.0002
**Momentum**	0.9	0.9	
**Weight decay**	0.0001	0.0001	0.0001
**Data augmentation**	RandomChoiceResize[(1333, x)] [2], RandomHorizontalFlip, Normalize	RandomChoiceResize[(1333, x)] [2], RandomHorizontalFlip, Normalize	RandomChoice[RandomChoiceResize(x, 1333), RandomCrop] [2], RandomHorizontalFlip, RandomColorJitter, Normalize
**Loss function**	L1 loss (reg), cross entropy (cls)	L1 loss (reg), cross entropy (cls)	L1 loss (reg), focal loss (cls), GIoU loss (bbox)
**Optimizer**	SGD	SGD	AdamW
**Scheduler**	StepLR	StepLR	MultiStepLR

[1] Backbones were pretrained on ImageNet.

[2] Here, x refers to the following scales: [(400, 500, 600), (480, 512, 544, 576, 608, 640, 672, 704, 736, 768, 800)].

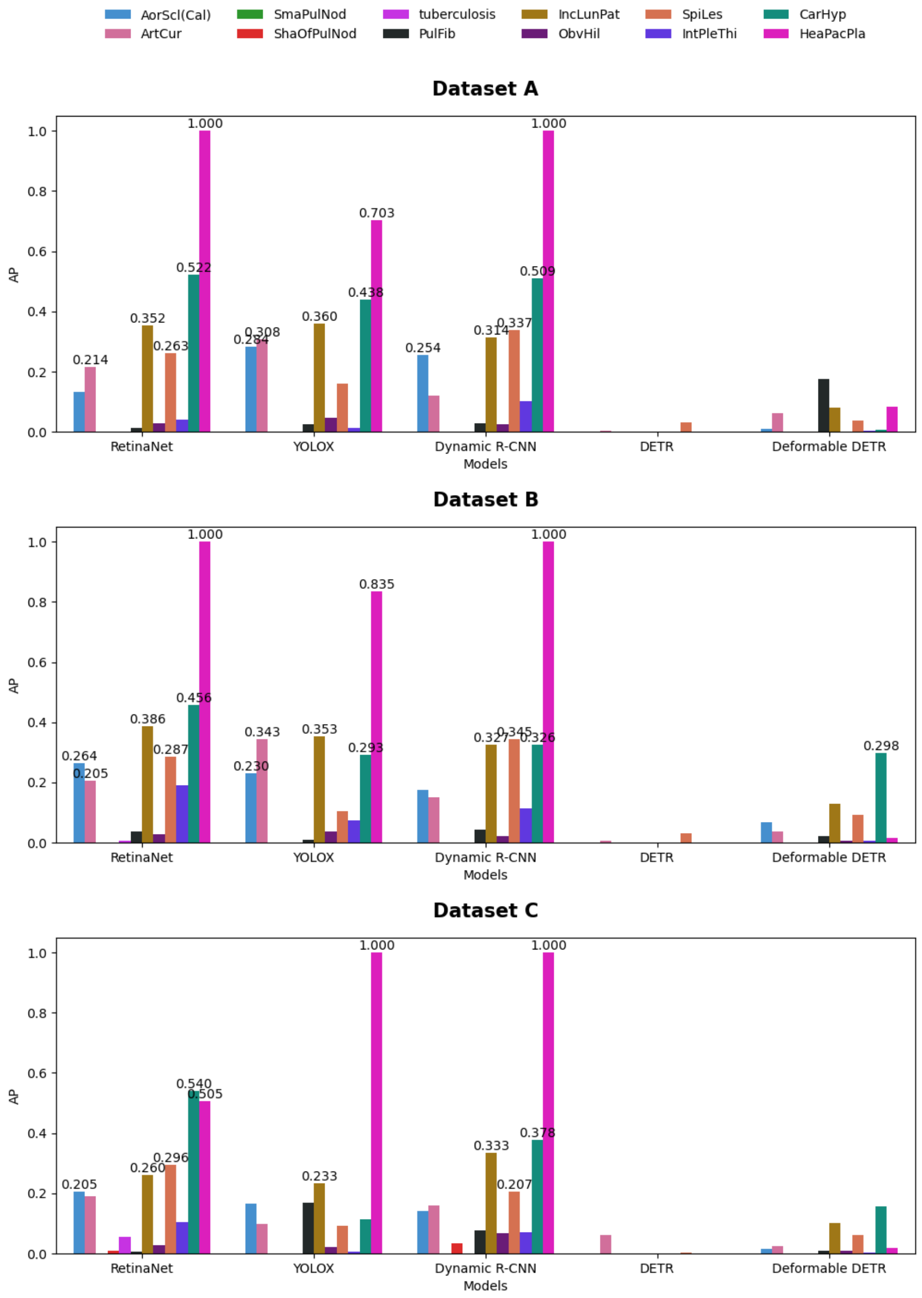

Figure S1. Disease-wise average precision (AP) values for Scheme 1.

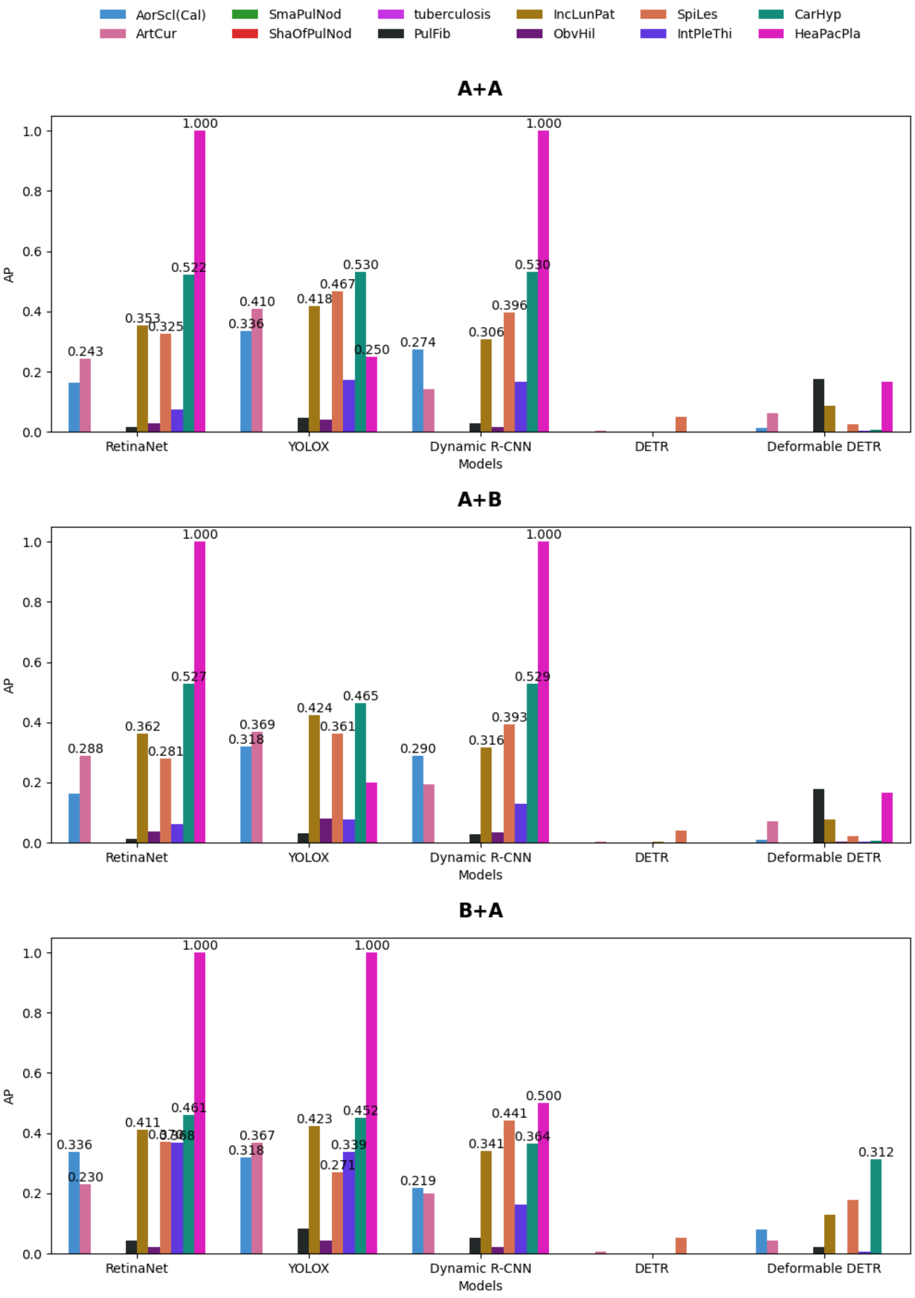

Figure S2. Disease-wise average precision (AP) values for Scheme 2.

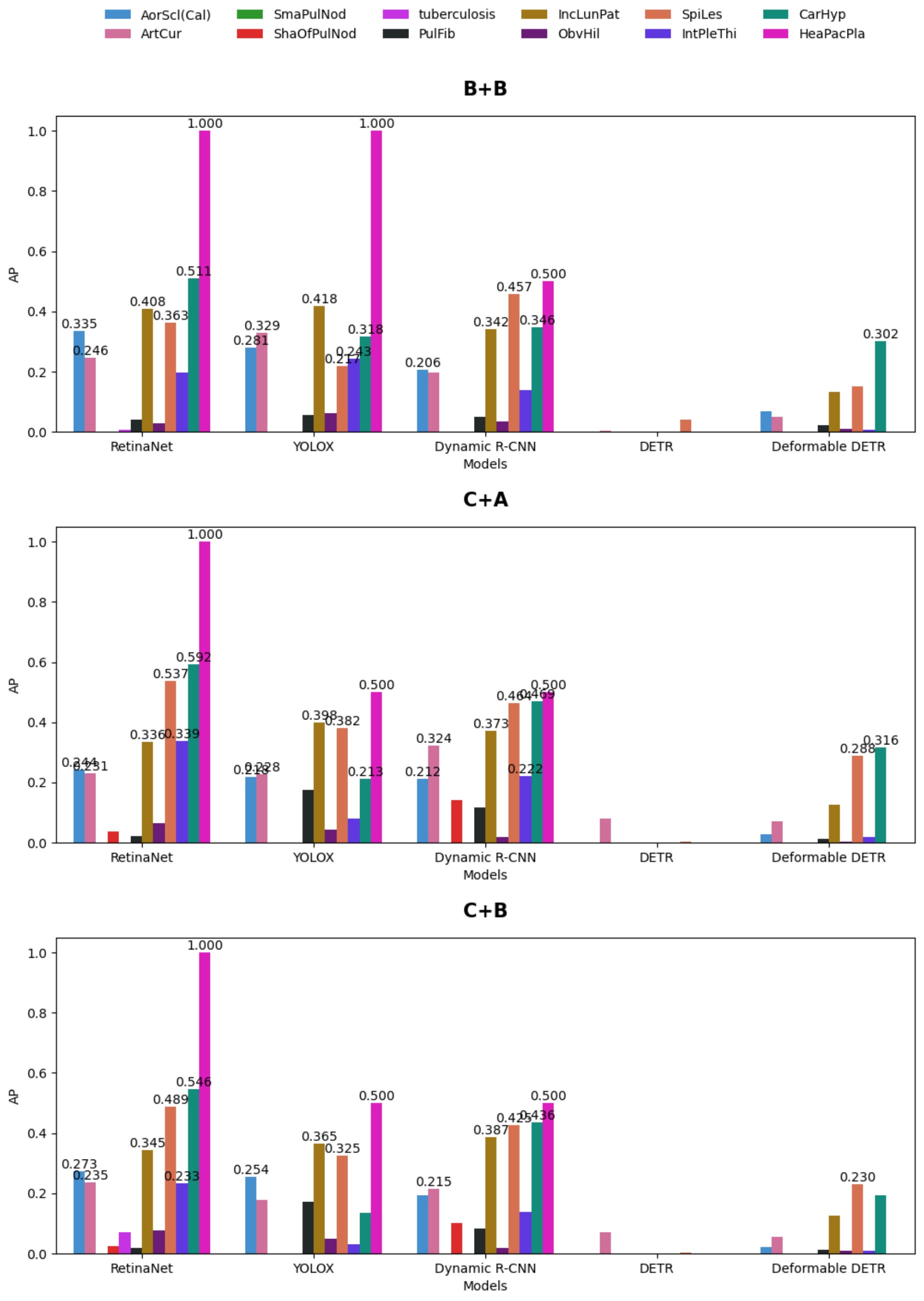

Figure S2 (cont.). Continued disease-wise average precision (AP) values for Scheme 2.

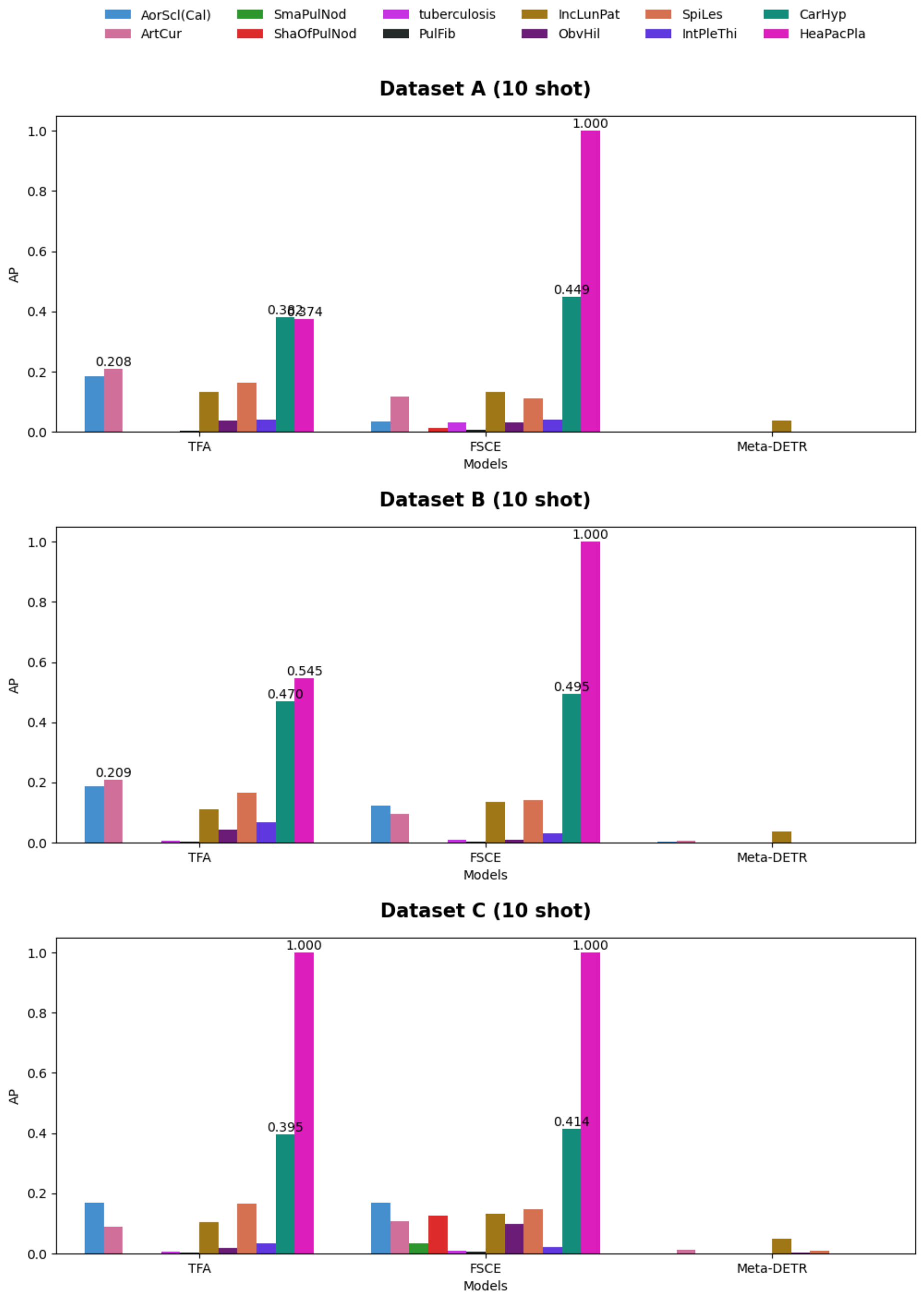

Figure S3. Disease-wise average precision (AP) values for Scheme 3.

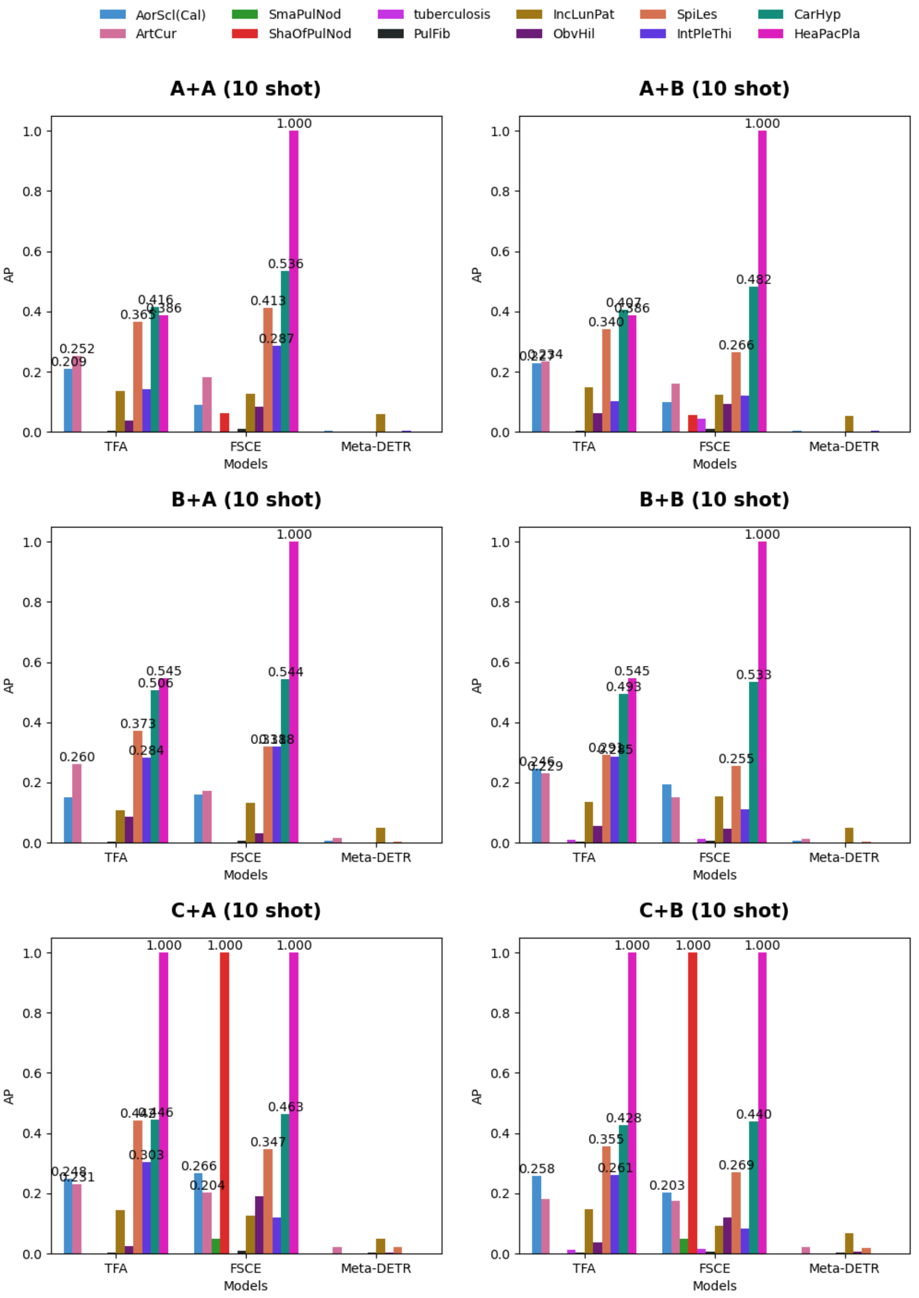

Figure S4. Disease-wise average precision (AP) values for Scheme 4.

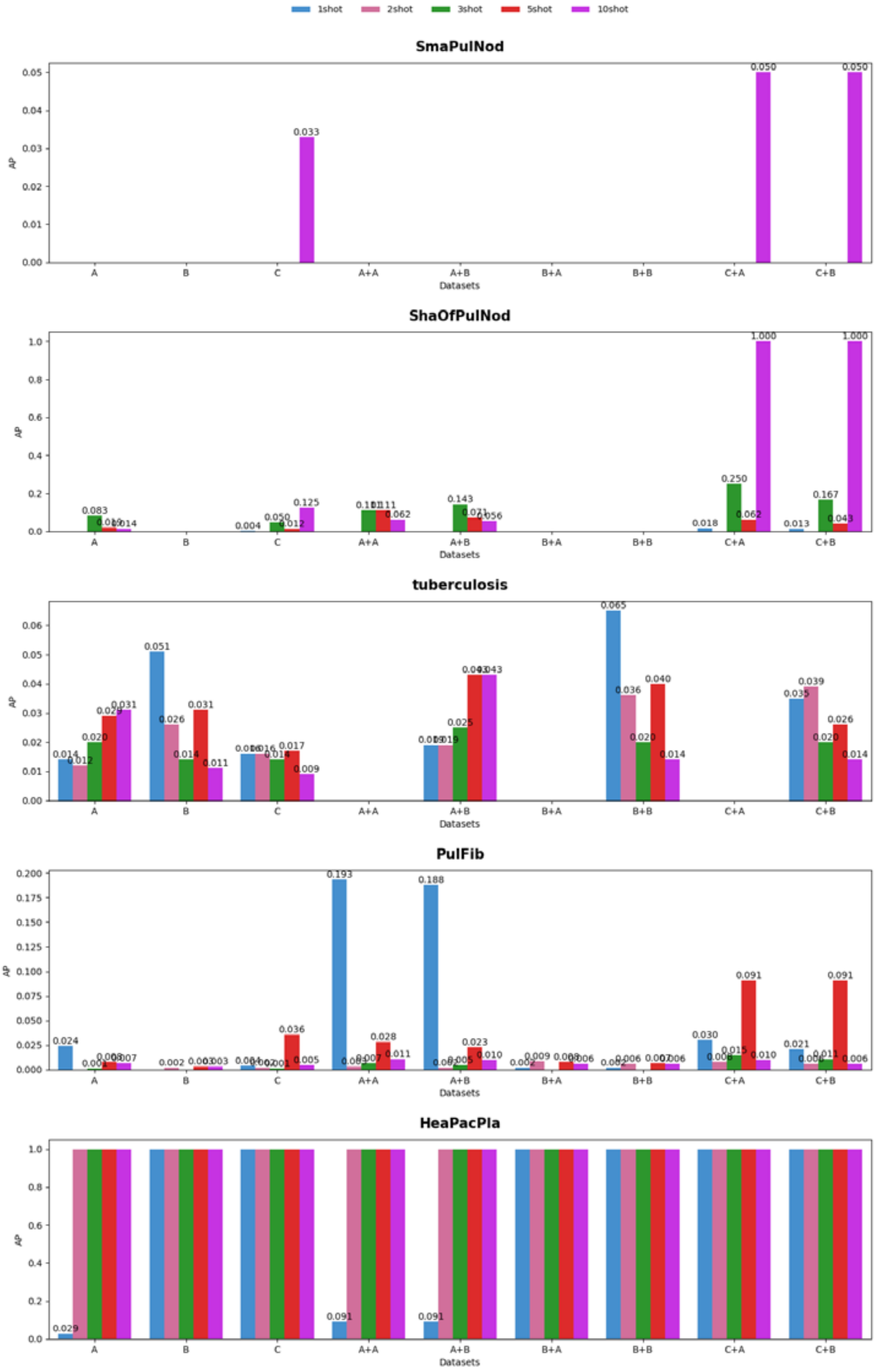

Figure S5. Novel class APs of FSCE for different shot settings.
